# Independent external validation and comparison of prevalent diabetes risk prediction models in a mixed-ancestry population of South Africa

**DOI:** 10.1186/s13098-015-0039-y

**Published:** 2015-05-09

**Authors:** Katya Masconi, Tandi E. Matsha, Rajiv T. Erasmus, Andre P. Kengne

**Affiliations:** Division of Chemical Pathology, Stellenbosch University, Cape Town, South Africa; Non-Communicable Diseases Research Unit, South African Medical Research Council, Cape Town, South Africa; Faculty of Health and Wellness Sciences, Cape Peninsula University of Technology, Cape Town, South Africa; Department of Medicine, Groote Schuur Hospital, University of Cape Town, Cape Town, South Africa

## Abstract

**Background:**

Guidelines increasingly encourage the use of multivariable risk models to predict the presence of prevalent undiagnosed type 2 diabetes mellitus worldwide. However, no single model can perform well in all settings and available models must be tested before implementation in new populations. We assessed and compared the performance of five prevalent diabetes risk models in mixed-ancestry South Africans.

**Methods:**

Data from the Cape Town Bellville-South cohort were used for this study. Models were identified via recent systematic reviews. Discrimination was assessed and compared using C-statistic and non-parametric methods. Calibration was assessed via calibration plots, before and after recalibration through intercept adjustment.

**Results:**

Seven hundred thirty-seven participants (27 % male), mean age, 52.2 years, were included, among whom 130 (17.6 %) had prevalent undiagnosed diabetes. The highest c-statistic for the five prediction models was recorded with the Kuwaiti model [C-statistic 0.68: 95 % confidence: 0.63–0.73] and the lowest with the Rotterdam model [0. 64 (0.59–0.69)]; with no significant statistical differences when the models were compared with each other (Cambridge, Omani and the simplified Finnish models). Calibration ranged from acceptable to good, however over- and underestimation was prevalent. The Rotterdam and the Finnish models showed significant improvement following intercept adjustment.

**Conclusions:**

The wide range of performances of different models in our sample highlights the challenges of selecting an appropriate model for prevalent diabetes risk prediction in different settings.

**Electronic supplementary material:**

The online version of this article (doi:10.1186/s13098-015-0039-y) contains supplementary material, which is available to authorized users.

## Background

Diabetes mellitus, type 2 diabetes in particular, is a growing epidemic worldwide with developing countries currently paying the highest toll [1]. In 2013 there were approximately 382 million individuals with type 2 diabetes, and this number will surge to approximately 592 million by 2035 [1]. This rapid rise of diabetes will result in an even greater and more profound burden which developing countries are not equipped to handle. Type 2 diabetes in developing countries is further characterized by a low detection rate with a high proportion of people being undiagnosed. Strategies are therefore needed for early detection and risk stratification such that treatment measures can be implemented to prevent the onset or delay the progression of related complications.

The use of multivariable risk prediction models has been advocated as practical and potentially affordable approaches for improving the detection of undiagnosed diabetes. Accordingly, guidelines, including those of the International Diabetes Federation, increasingly promote the use of reliable, simple and practical risk scoring systems or questionnaires and derivatives for diabetes risk screening around the world [2, 3]. During the last two decades, numerous diabetes prediction models have been developed. However, only a few models have been externally validated, and generally not in developing countries [4, 5]. Consequently, many developing countries have to rely on prediction models developed in other populations and not necessarily validated in their context. However, issues relating to differences in case-mix across populations, inherent to the development of models, can severely affect the applicability of a model in different settings [6, 7].

This study aimed to validate and compare the performance of selected common models for predicting prevalent undiagnosed diabetes based upon non-invasively measured predictors, in mixed ancestry South Africans.

## Methods

### Study population and design of study

The Cape Town Bellville-South study data served as the basis for models validation [8]. Bellville-South is located within the Northern suburbs of Cape Town, South Africa and is a traditionally a mixed-ancestry township formed in the late 1950s. According to the 2001 population census, its population stands at approximately 26,758 with 80.48 % (21,536) consisting of the mixed ancestry individuals [22]. The study was approved by the Ethics Committee of the Cape Peninsula University of Technology (CPUT/HW-REC 2008/002 and CPUT/HW-REC 2010) and Stellenbosch University (N09/05/146).

The Bellville South Study was a cross-sectional study conducted from mid-January 2008 to March 2009 (cohort 1), and from January 2011 to November 2011 (cohort 2). The target population for this study were subjects ≥ 35 y. Using a map of Bellville South obtained from the Bellville municipality, random sampling was approached as follows: first, the area was divided into six strata; second, within each strata the streets were classified as short (≤22 houses), medium (23–40 houses) and long (≥40 houses) streets based on the number of houses. Two of each respective streets were randomly selected from each strata. In those instances where the numbers of houses were too few, a short or a medium street was randomly selected and added to such a stratum. The result was a total of 16 short streets representing approximately 190 houses, 15 medium (approximately 410 houses) and 12 long streets (approximately 400 houses). From the selected streets, all household members meeting the selection criteria were invited to participate in the study. One thousand subjects who met the criteria were approached and 642 participated in the study. In addition, community authorities requested that willing participants outside the random selection area should benefit from the study. Therefore volunteers (304 in 2008–2009 [cohort 1), and 308 in 2011 [cohort 2]) from the same community, but who were not part of the randomly selected streets or did not meet the age criteria, were also included.

### Recruitment strategy

Information regarding the project was disseminated to residents through the local radio station, community newspaper, brochures and fliers; the latter bearing information about the project and distributed through school children and taxis by the recruitment team. Additionally, a ‘road show’ strategy that involved a celebrity suffering from diabetes from the same community was also used, especially in the targeted streets. Recruited subjects were visited by the recruitment team the evening before participation and reminded of all the survey instructions. These included overnight fasting, abstinence from drinking alcohol or consumption of any fluids in the morning of participation. Since the participants were required to bring in an early morning mid-stream urine sample, they were provided with a sterile container as well as instructions on how to collect the sample. Furthermore, participants were encouraged to bring along their medical/clinic cards and/or medication they were currently using.

### Identification of prediction models

Existing prediction models were obtained from a systematic review by Brown *et al.* [9]. The search strategy from Brown’s paper was re-run in PubMed for the time-period up to April 2014, to identify possible new models. The following string search was used, as per Brown *et al.*: ((“type 2 diabetes” OR “hyperglycaemia” OR “hyperglycemia”) AND (“risk scores)).” Selected models were only those developed to predict the presence of undiagnosed diabetes. We focused on models developed using non-invasively measured predictors which were available in the Bellville-South cohort database. Models were excluded if they were developed for male and female individual separately.

### Outcome and predictors’ definition and measurements

The main outcome was newly diagnosed type 2 diabetes from the standard oral glucose tolerance test (OGTT), applying the World Health Organisation (WHO) criteria (i.e. fasting plasma glucose ≥ 7.0 mmol/L and/or 2 h plasma glucose ≥ 11.1 mmol/L) [10]. At the baseline evaluation conducted between 2008 and 2011, participants received a face-to-face interview administered by trained personnel to collect data on personal and family history of diabetes mellitus, cardiovascular disease (CVD) and treatments; habits including smoking, alcohol consumption, physical activity and diet; demographics and education.

Clinical measurements included: height, weight, hip and waist circumferences and blood pressure (BP). BP measurements used a semi-automatic digital blood pressure monitor (Rossmax MJ90, USA) on the right arm, in sitting position, after a 10 min rest. The lowest value from three consecutive measurements 5 min apart was used in the current analysis. Weight to the nearest 0.1 kg was determined on a Sunbeam EB710 digital bathroom scale, with each subject in light clothing, without shoes and socks. Height to the nearest centimetre was measured with a stadiometer, with subjects standing on a flat surface. Body Mass Index (BMI) was calculated as weight per square meter (kg/m^2^).

Blood samples were collected and processed for a wide range of biochemical markers. Plasma glucose was measured by enzymatic hexokinase method (Cobas 6000, Roche Diagnostics, USA). High density lipoprotein cholesterol (HDL-c) and triglycerides (TG) were estimated by enzymatic colorimetric methods (Cobas 6000, Roche Diagnostics, USA).

### Assessment of model performance

The original selected models were validated for the overall data and subsets using the formulas, without any recalibration. The predicted probability of undiagnosed diabetes for each participant was computed using the baseline measured predictors. The performance was expressed in terms of discrimination and calibration. Discrimination describes the ability of the model’s performance in distinguishing those at a high risk of developing diabetes from those at low risk [11]. The discrimination was assessed and compared using concordance (C) statistic and non-parametric methods [12].

Calibration describes the agreement between the probability of the outcome of interest as estimated by the model, and the observed outcome frequencies [13]. It was assessed graphically by plotting the predicted risk against the observed outcome rate. The agreement between the expected (E) and observed (O) rates (E/O) was assessed overall and within pre-specified groups of participants. The 95 % confidence intervals for the expected/observed probabilities (E/O) ratio were calculated assuming a Poisson distribution [14]. We also calculated 1) the Yates slope, which is the difference between mean predicted probability of type 2 diabetes for participants with and without prevalent undiagnosed diabetes, with higher values indicate better performance; and 2) the Brier score, which is the squared difference between predicted probability and actual outcome for each participant with values ranging between 0 for a perfect prediction model and 1 for no match in prediction and outcome [11, 13]. To determine optimal cut-off for maximising the potential effectiveness of a model, the Youden’s J statistic (Youden’s index) was used to determine the best threshold [15], with sensitivity, specificity and percentage of correctly classified individuals determined for each threshold. The main analysis was done for the overall cohort and for subgroups defined by sex, age (<60 vs. ≥60 years) and BMI (<25 kg/m^2^ vs. ≥25 g/m^2^).

### Sensitivity analysis

To improve performance and eliminate differences in diabetes prevalence between the development population and the test population, models were recalibrated to the test-population-specific prevalence using intercept adjustment [16]. The correction factor calculated is based on the mean predicted risk and the prevalence in the validation set and is the natural logarithm of the odds ratio of the mean observed prevalence and the mean predicted risk [16]. To assess the potential effect on model performance of validation studies from complete case analysis, we also assess the discrimination of model across five datasets after application of multiple data imputation procedures to fill missing data.

## Results

### Identification of prediction models

**Fig. 1:**
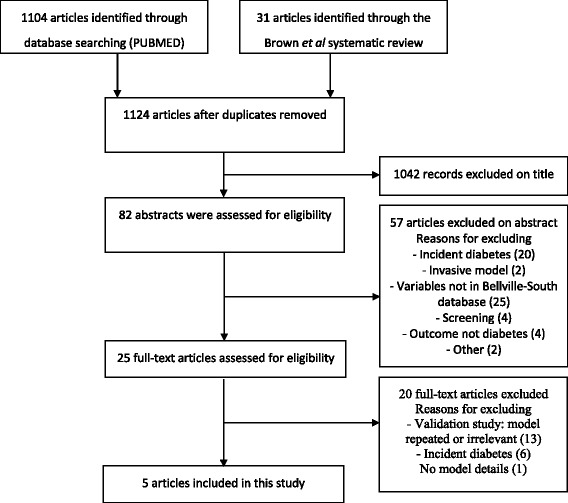
Flow diagram of selected studies

Five non-invasive prevalent diabetes prediction models were selected for validation following the screening process; the Cambridge Risk Score [17], Kuwaiti Risk Score [18], Omani Diabetes Risk Score [19], Rotterdam Predictive Model 1 [20] and the simplified Finnish Diabetes Risk Score [21] (Fig. 1). Table 1 summarizes the models’ characteristics. All models included age as a predictor, while a range of other predictors were variably combined in models. These included: sex, BMI, use of antihypertensive medication, family history of diabetes, waist circumference, past or current smoking and the use of corticosteroids. Additional 1: Table S1 comprises of the full equations for each of the models.

**Table 1:** Overview of the included prevalent diabetes risk prediction models and their performance for the original model and the intercept adjusted model

	Incident diabetes risk models										
Description	Cambridge risk score		Kuwaiti risk score		Omani risk score		Rotterdam predictive model 1		Simplified Finnish risk score		Bellville South
Authors	Griffin *et al.* [17]		Al Khalaf *et al.* [18]		Al-Lawati & Tuomilehto [19]		Baan *et al.* [20]		Bergmann *et al.* [21]		-
Year published	2000		2008		2007		1999		2007		-
Country	UK		Kuwaiti		Oman		Netherlands		Germany		South Africa
Validation	External [23-28, 35]		None		External [28]		External [23, 27, 29]		External [23, 27, 28]		-
Sample size	1077		460		4881		1016		526		737
Type of study	Cross-sectional		Cross-sectional		Cross-sectional		Cohort		Cohort		Cohort
Age range	40 – 79		20 – >40 (36.2)		20 – 80		55 – 75		41 – 79		15–95
Population	Caucasian		Arab		Arab		Caucasian		Caucasian		Mixed ancestry
Diagnosis of diabetes	FBG ≥ 7.0 mmol/l; 2 h glucose ≥ 11.1 mmol/l		FBG ≥ 7.0 mmol/l; Random glucose ≥ 11.1 mmol/l		FBG ≥ 7.0 mmol/l; 2 h glucose ≥ 11.1 mmol/l		FBG ≥ 7.0 mmol/l; 2 h glucose ≥ 11.1 mmol/l		FBG ≥ 7.0 mmol/l; 2 h glucose ≥ 11.1 mmol/l		FBG ≥ 7.0 mmol/l; 2 h glucose ≥ 11.1 mmol/l
Development C-statistic	0.80 (0.68 – 0.91)		0.82 (NS)		0.83 (0.82 – 0.84)		0.68 (0.64 – 0.72)		0.75 (0.68 – 0.81)		-
Predictors											
*Age*	Yes		Yes		Yes		Yes		Yes		Yes
*Sex–*	Yes		No		No		Yes		Yes		Yes
*BMI*	Yes		No		Yes		Yes		Yes		Yes
*Use of HTN drugs*	Yes		Yes		No		Yes		Yes		Yes
*Family history*	Yes		Yes		Yes		No		Yes		Yes
*WC*	No		Yes		Yes		No		No		Yes
*Smoking*	Yes		No		No		No		Yes		Yes
*Corticosteroids*	Yes		No		No		No		Yes		Yes
*Systolic/diastolic*	No		No		Yes		No		No		Yes
Performance	Original	Adjusted	Original	Adjusted	Original	Adjusted	Original	Adjusted	Original	Adjusted	
E/O (95 % CI)	1.81 (1.09–2.52)	1.22 (0.61–1.83)	0.72 (0.40–1.12)	0.94 (0.47–1.41)	1.28 (0.63–1.93)	1.06 (0.47–1.66)	0.54 (0.50–1.04)	0.98 (0.91–1.05)	0.26 (0.13–0.39)	0.89 (0.51–1.26)	−
Brier score	0.193	0.160	0.141	0.143	0.164	0.157	0.147	0.140	0.157	0.143	−
Yates slope	0.379	0.379	0.496	0.496	0.392	0.392	0.971	0.971	0.491	0.491	−
C-statistic (95 % CI)	0.67 (0.62–0.72)	−	0.68 (0.63–0.73)	−	0.66 (0.61–0.70)	−	0.64 (0.59–0.69)	−	0.67 (0.62–0.71)	−	−
Optimal threshold	0.29	0.16	0.13	0.18	0.12	0.09	0.20	0.18	0.02	0.08	−
Sensitivity	65	65	61	61	85	85	57	57	75	75	−
Specificity	61	61	63	63	42	42	65	65	48	48	−
Correctly classified	62	62	63	63	50	50	64	64	53	53	−

*95 % CI 95 % confidence interval, *BMI* body mass index, *DM* diabetes mellitus, *E/O* ratio expected/observed event rate, *FBG* fasting blood glucose, *HTN* hypertension, *OGTT* 2 h post load oral glucose tolerance test, *UK* United Kingdom, *WC* waist circumference

### Participants’ characteristics

A total of 1256 participants were examined in the Bellville South studies, including 173 with a history of diagnosed diabetes who were excluded. A further 346 participants were excluded for missing data on predictors or outcome variable. Therefore the final dataset comprised of 737 participants, of whom 580 (78.70 %) were female. In the Additional file 2: Table S2, we compare the profile of participants in the final sample vs. that of participants excluded for missing data. Excluded participants comprised more men (27.2 vs. 21.3 %, p = 0.012), were more likely to display a better lifestyle profile for alcohol intake (18.8 % vs. 28.1 %, p <0.001), smoking (31.8 % vs. 43.8 %, p < 0.001), lower family history of diabetes (all p ≤0.001), higher systolic blood pressure (126 vs. 123 mmHg, p = 0.009) and lower triglycerides (1.4 vs. 1.5 mmol/l, p = 0.043); although absolute differences were mostly clinically trivial.

The baseline profile for men and women included in the study is described in Table 2. The mean baseline age was 51.2 years overall, and 53.5 and 52.1 years, respectively in men and women (p = 0.311). The BMI (p < 0.001) waist circumference (p = 0.024) and fasting blood glucose (p = 0.036) were significantly higher in women, while smoking (p <0.001) and alcohol consumption (p <0.001) were frequent among men.

**Table 2:** Characteristics comparison of participants with valid data between male and female

	Male (157)	Female (580)	p-value	Overall (737)
Prevalent undiagnosed DM (%)	22 (14.0)	108 (18.6)	0.220	130 (17.3)
Age (years)	53.5 (15.0)	52.1 (14.3)	0.311	52.2 (14.5)
Body mass index (kg/m^2^)	25.5 (5.8)	29.6 (7.0)	<0.001	29.4 (7.1)
Waist circumference (cm)	92.5 (15.2)	95.6 (14.7)	0.024	95.9 (14.9)
Hypertensive medication (%)	43 (27.4)	208 (35.9)	0.059	251 (34.1)
Smoking status (% smoking)	88 (56.1)	235 (40.5)	<0.001	323 (43.8)
Systolic blood pressure (mmHg)	124.3 (16.6)	121.6 (19.2)	0.077	122.0 (18.7)
Diastolic blood pressure (mmHg)	75.6 (11.1)	74.7 (12.1)	0.365	74.7 (11.9)
Height (m)	1.7 (0.1)	1.6 (0.1)	<0.001	1.6 (0.1)
Mother having diabetes (%)	17 (10.8)	92 (15.9)	0.147	109 (14.8)
Father having diabetes (%)	14 (8.9)	44 (7.6)	0.702	58 (7.9)
Sister having diabetes (%)	12 (7.6)	80 (13.8)	0.053	92 (12.5)
Brother having diabetes (%)	9 (5.7)	49 (8.5)	0.340	58 (7.9)
Fasting blood glucose (mmol/L)	5.4 (1.4)	5.7 (2.0)	0.036	5.8 (1.9)
HDL (mmol/L)	1.2 (0.4)	1.3 (0.3)	0.136	1.3 (0.3)
Weight (kg)	72.3 (16.4)	73.9 (17.7)	0.290	74.1 (17.5)
Ever consumed alcohol (%)	116 (73.9)	240 (41.4)	<0.001	356 (48.3)
Current drinking (%)	80 (51.0)	127 (21.9)	<0.001	207 (28.1)
Using Corticosteroid use (%)	1 (0.6)	4 (0.7)	>0.99	5 (0.7)
Triglyceride (mmol/L)	1.4 (0.9)	1.4 (0.9)	0.836	1.4 (0.9)

### Prediction of prevalent undiagnosed diabetes in the overall sample

**Fig. 2:**
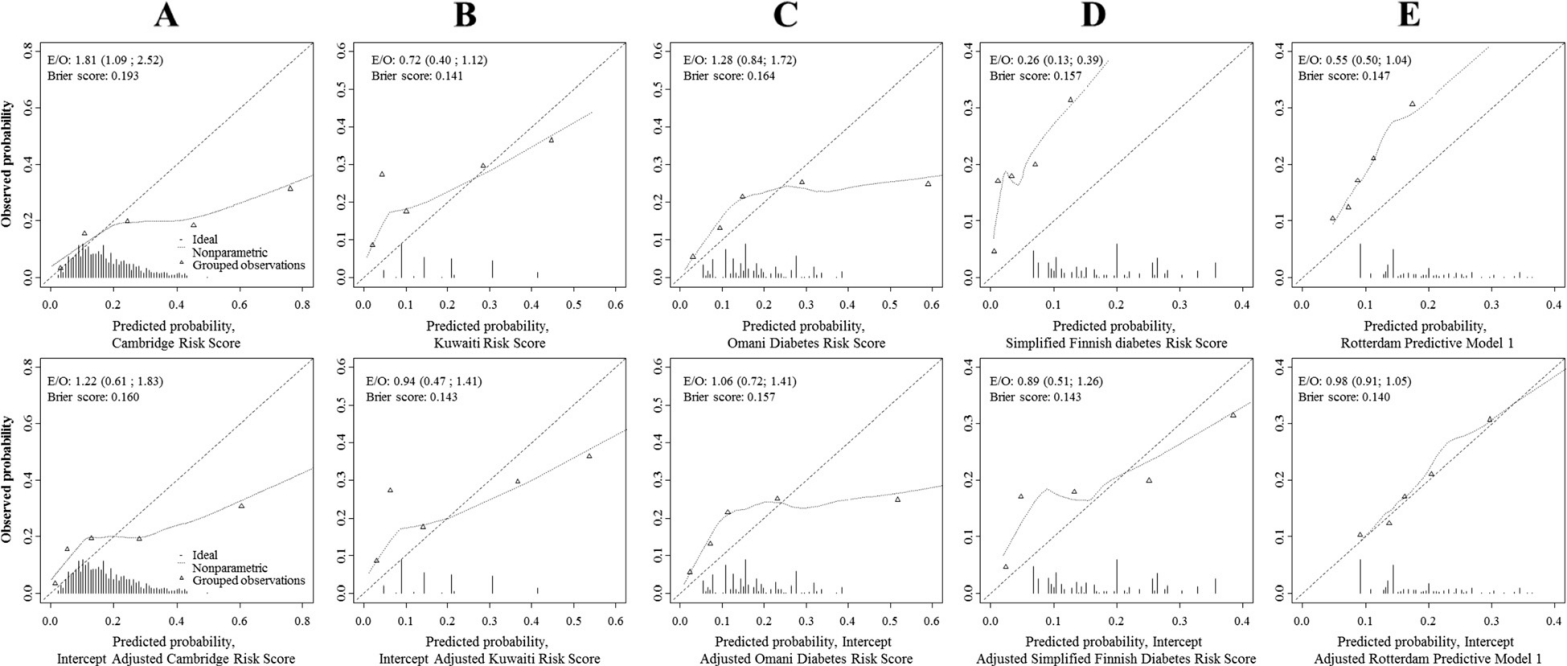
Calibration curves in the overall cohort for the models before (upper panel) and after the intercept adjustment (lower panel). **A** Cambridge Risk Score, **B** Kuwaiti Risk Score, **C** Omani Diabetes Risk Score, and **D** Simplified Finnish Diabetes Risk Score and **E** Rotterdam Predictive Model 1. Calibration describes the agreement between the probability of undiagnosed diabetes as estimated by the model and the recorded frequencies of the outcome. The ideal calibration is graphically represented by the dotted diagonal line at 45°. Participants are grouped into percentiles across increasing predicted risk. The vertical lines at the bottom of the graph depict the frequency distribution of the calibrated probabilities of diabetes. E/O, expected/observed ratio

A total of 130 participants (17.6 %) had prevalent undiagnosed diabetes. This prevalence was similar in men vs. women (14 % vs. 18.6 %, p = 0.220) (Table 2). Table 1 and Additional file 1: Table S1 shows the discrimination for the selected prediction models in their original form in the overall sample. Discrimination was modest-to-acceptable and similar between models, with C-statistics (95 % CI) ranging from 0.64 (0.59–0.69) for the Rotterdam model to 0.68 (0.63–0.73) for the Kuwaiti model (all p > 0.05 for c-statistics comparison; Additional file 3: Table S3). At the total population level, the absolute risk of prevalent diabetes was acceptably estimated by the Omani model, overestimated by 81 % (9–152 %) by the Cambridge model, underestimated by 74 % (61–87 %) by the Finnish model and marginally underestimated by the Kuwaiti and Rotterdam models (Table 1). The calibration curves are shown in Fig. 2 and supplemental Fig. 2. There was a systematic risk underestimation across the continuum of predicted probability by the Finnish and Rotterdam models, a selective upper strata risk overestimation by the Cambridge and Omani models, and a combination of both lower strata risk underestimation and upper strata risk overestimation by the Kuwaiti model. Comparison of the C-statistics from the development study and the models’ performance in this population shows a drop in performance of all the models. Other performance measures are shown in Table 1.

### Prediction of prevalent undiagnosed diabetes in subgroups

The performance of the original models across subgroups was parallel to that in the overall dataset (Table 3). When comparing patterns of predictions across complementary subgroups, only stand-alone differences were seen in performance for a subgroup, which was not carried through all performance measures. Estimates of C-statistics were broadly similar across complementary subgroups, except for the Omani and Finnish models across BMI subgroups, whereby lower estimates were always found in the overweight/obese subgroup. The pattern of the overall calibration (E/O) across complementary subgroups varied substantially across models. For instance, across gender subgroups, the overall diabetes risk was acceptably and equally predicted by the Omani model, equally underestimated by the Kuwaiti and Finnish models, equally overestimated by the Cambridge model, but acceptably estimated in men and underestimated in women by the Rotterdam model (Table 3). Other performance measures across subgroups are shown in Table 3.

**Table 3:** Discrimination and calibration statistics for diabetes risk model performance in subgroups of participants by gender, age and body mass index (BMI)

Models		Male	Female	Age < 60 years	Age ≥ 60 years	BMI < 25 kg/m^2^	BMI ≥ 25 kg/m^2^
Cambridge Diabetes Risk Score [17]	E/O (95 % CI)	2.30 (1.21−3.37)	1.71 (1.00−1.41)	1.57 (0.71−2.44)	2.10 (1.51−2.69)	1.08 (0.55−1.61)	1.96 (1.30−2.63)
	Brier score	0.195	0.192	0.151	0.282	0.102	0.230
	Yates slope	0.373	0.384	0.368	0.384	0.450	0.368
	C-statistic (95 % CI)	0.67 (0.56−0.78)	0.67 (0.62−0.73)	0.66 (0.60−0.72)	0.65 (0.56−0.73)	0.69 (0.58−0.79)	0.64 (0.59−0.70)
Kuwaiti Risk Score [18]	E/O (95 % CI)	0.73 (0.40−1.06)	0.72 (0.34−1.10)	0.73 (0.37−1.10)	0.71 (0.32−1.11)	0.33 (0.20−0.46)	0.81 (0.43−1.19)
	Brier score	0.112	0.149	0.121	0.186	0.097	0.159
	Yates slope	0.588	0.468	0.476	0.449	0.890	0.468
	C-statistic (95 % CI)	0.70 (0.58−0.82)	0.67 (0.61−0.72)	0.67 (0.61−0.74)	0.65 (0.57−0.73)	0.61 (0.51−0.72)	0.66 (0.60−0.71)
Omani Diabetes Risk Score [19]	E/O (95 % CI)	1.33 (0.45−2.20)	1.32 (0.65−2.00)	1.26 (0.53−1.99)	1.40 (0.60−2.20)	1.16 (0.41−1.92)	1.36 (0.71−2.01)
	Brier score	0.137	0.173	0.140	0.221	0.096	0.194
	Yates slope	0.347	0.399	0.393	0.296	0.620	0.304
	C-statistic (95 % CI)	0.62 (0.49−0.74)	0.66 (0.61−0.71)	0.66 (0.60−0.71)	0.60 (0.52−0.68)	0.71 (0.61−0.82)	0.61 (0.56−0.67)
Rotterdam Predictive Model 1 [20]	E/O (95 % CI)	0.84 (−0.38−2.06)	0.48 (0.45−0.93)	0.52 (0.44−0.96)	0.49 (0.39−0.88)	0.72 (0.34−1.06)	0.51 (0.45−0.96)
	Brier score	0.117	0.155	0.125	0.199	0.096	0.168
	Yates slope	0.913	1.154	1.135	0.838	0.791	0.886
	C-statistic (95 % CI)	0.62 (0.49−0.75)	0.66 (0.60−0.72)	0.62 (0.55−0.69)	0.61 (0.52−0.69)	0.61 (0.50−0.72)	0.63 (0.57−0.69)
Simplified Finnish Diabetes Risk score [21]	E/O (95 % CI)	0.22 (0.09−0.35)	0.32 (0.18−0.45)	0.34 (0.18−0.50)	0.26 (0.14−0.37)	0.11 (0.06−0.15)	0.34 (0.21−0.48)
	Brier score	0.128	0.162	0.128	0.213	0.103	0.176
	Yates slope	0.538	0.591	0.487	0.608	1.345	0.562
	C-statistic (95 % CI)	0.70 (0.59−0.81)	0.66 (0.60−0.71)	0.64 (0.58−0.71)	0.67 (0.60−0.75)	0.77 (0.69−0.86)	0.62 (0.57−0.68)

### Performance of the intercept adjusted models

As expected, intercept adjustment yielded acceptable agreement between predicted and observed prevalent diabetes rates at the total population level. A perfect agreement was also observed across the continuum of the predicted probability by the updated Rotterdam model. However, despite some attenuation, selective upper strata risk overestimations were apparent for other models.

### Model performance at the optimal threshold

The performances of models at the optimal thresholds are shown in Table 1. As expected, the optimal threshold probability for our sample varied across models and for the same model between the original and intercept adjusted versions. The sensitivity at the optimal threshold ranged from 61 % for the Kuwaiti model to 85 % with the Omani model, the specificity from 42 % (Omani model) to 65 % (Rotterdam model), and the proportion of participants correctly classified from 50 % (Omani model) to 64 % (Rotterdam model).

### Model performance after multiple imputation of missing data

The discrimination (c-statistic) of models across five datasets obtained after multiple imputation of missing data was very similar: 0.69 (0.64–0.73) for the Cambridge model, 0.69 (0.65–0.74) for the Kuwaiti model, 0.65 (0.61–0.69) for the Omani model, 0.65 (0.60–0.69) for the Rotterdam model and 0.66 (0.62–0.70) for the Finnish model. The values were also very similar to those from the validation of models on dataset comprising only participants with complete data (Table 1).

## Discussion

To our knowledge, this is the largest and most comprehensive validation study of prevalent diabetes prediction models in a sub-Saharan African population. In the Bellville South cohort, the selected existing prediction models based upon non-invasive measured predictors had modest-to-acceptable discriminatory ability to predict prevalent undiagnosed diabetes, both overall and within subgroups. Simple intercept adjustment had mixed effect on the calibration performance of the models, while none of the models was significantly better than other models to be uniquely recommended for use in this setting. At the optimal probability thresholds, the best performing model would correctly classify only about 2/3^rd^ of the population, indicating the existing scope for further improving the models’ performance in this setting.

The need for diabetes screening programs is imperative in the reduction of the worldwide burden of complications from diabetes in undiagnosed individuals. In view of the large and continuously growing burden of diabetes the Centre for Disease Control strongly advocates for diabetes screening programs. In its most recent guidelines for type 2 diabetes screening and diagnosis, the International Diabetes Federation has recommended that each health service should decide on programs to detect undiagnosed diabetes based on the prevalence and the resources available in that region [3]. In areas with limited care, such as developing countries, the detection programs are suggested to be opportunistic and should be limited to high-risk individuals. The World Health Organization African region promotes the screening of at-risk individuals in Africa in healthcare settings and social gatherings [22]. Risk assessment scores are feasible and cost-effective and can be considered, but applicability must be certain, with the required tests available in the area and the validation of that risk score in the population.

With the exception of the Kuwaiti model [18], all other models assessed in our study have been validated externally. The most validated appeared to be the Cambridge model [17], with c-statistics ranging from 0.67 to 0.83 across validation studies [23-27]. With a c-statistic of 0.67 in the Bellville South data set, the Cambridge model performance in this population fell to the bottom end of other validation study results. Similarly, the Finnish model’s discrimination performance (c-statistic: 0.67) also compared with lower c-statistic’s from validation studies [23, 27, 28]. The Rotterdam model mirrored the validation study results (0.64 vs. 0.63–0.65) [23, 27, 29], while the Omani model underperformed (c-statistic: 0.66) when compared to the only validation study the authors are aware of (c-statistic: 0.72) [28].

Through an attempt to improve calibration with simple intercept adjustment, the E/O ratios for all models were improved. Despite the expected decision that no model was ready for immediate implementation, the Rotterdam Predictive Model 1 showed the best improvement in calibration following this adjustment. A review by Brown *et al.* in 2012 [9] of 17 undiagnosed Type 2 diabetes risk scores, which included all five models discussed here, determined that performance was not associated to the number of predictors in the model. Overall, validation studies showed a drop in model performance when tested in a new population, with the Rotterdam model having the lowest validation performance range, when compared to the other models. This was echoed in our results for the original Rotterdam model validation. The possible reasons to explain the drop in the performance of diabetes prediction models in new population, some of which apply to our study, have been extensively discussed elsewhere [30].

At the optimal probability threshold, the models tested in our study would at best correctly detect two-thirds of participants, with diagnostic performance mostly similar to those from published studies [25, 30]. This indicates the existing scope for improving the performance of diabetes prediction models in our setting. This could be done by adopting or developing models enriched with predictors to improve the predictive accuracy. Such an approach however, has to be balanced against the fact that the number of predictors and the complexity and cost of their measurements are severe limitations for their uptake in routine practice [30]. What is probably needed the most in resources limited settings like Africa is evidence to confirm that the introduction of diabetes prediction models in routine practice will improve early detection of diabetes by healthcare practitioners, and the outcome of those diagnosed with diabetes in the long run.

The results of this study were strengthened by the diagnosis of diabetes based on OGTT, thus limiting the risk of misclassification. The age distribution was wide, including a vast majority of the high-risk population. A potential limitation of the study was the exclusion of some risk scores due to the necessary information being unavailable. The fewer number of males in the final dataset could have played a role in the performance of the models, owing to the significant difference between the genders in BMI, a predictor in four out of the five models. No power estimation was done, in the absence of consensus methods for sample size estimation in model validation studies. However, studies have suggested that at least 100 events and 100 non-events were the minimum required samples for external validation studies [31]. These requirements were largely met in our main analysis. Our study participants comprised a subset of randomly selected individuals and subset of self-selected participants from the same community. In the absence of any influence on participants’ selection of a prior knowledge of the association between relevant study outcomes and predictors included in tested model, any differential effect of the sample selection strategy on the discriminatory performance of tested models, is very unlikely. The prevalence of screen-detected diabetes in our randomly selected participants alones has been estimated to be 18.1 % [32], which is very close to the 17.6 % found in combined sample, suggested the absence of a differential effect on the calibration performance of models. The total number of participants with screen-detected diabetes in the combined sample precluded reliable stratified analyses to investigate and confirm the assumptions above. Finally, a substantial number of participants were excluded from the main analyses due to missing data on predictors included in models or on the status for prevalent undiagnosed diabetes. However, participants with complete data were mostly similar to those with missing data, particularly regarding the distribution of key predictors included in models such as age, gender and measures of adiposity. Therefore, differential effect on the model performance of validation based on complete case analyses, is very unlikely. Indeed, in sensitivity analysis, the discriminatory performance of models was very similar across multiple imputed datasets, and not appreciable different from the performance based on complete case analysis. Furthermore, variables with high frequency of missingness were likely to be those that are very difficult to accurately measure in routine setting like family history of diabetes, and therefore, less indicated for uncritical inclusion in models for predicting diabetes across settings [33, 34].

## Conclusions

Our findings highlight the performance variation of models differs across different populations, particularly calibration. This low performance can be explained by the obvious lack of transportability due to the differences in development and validation population characteristics and the affect case-mix difference has on model performance. With no model development in the mixed ancestry population of South Africa, selection of generalizable models for validation was limited. There is a great clinical need for a unique, robust and convenient tool for identifying undiagnosed diabetes and predicating future diabetes quicker and more economically in this South African population. Through efficient application of prediction models’ improvement procedures, the final model would improve risk assessment specific to this community. With no acceptable validated model, unique model development is possibly the best way forward.

## Additional files

Additional file 1: Table S1. Full equation for risk models to predict prevalent undiagnosed diabetes as applied to the Bellville South cohort. Additional file 2: Table S2. Characteristics comparison of participants with valid and missing data. Additional file 3: Table S3. Discrimination values and 95 % confidence intervals for selected models and the comparison of the discrimination between each model, expressed using p-value (<0.05 significant).
