# Supplementary material for: Independent external validation and comparison of prevalent diabetes risk prediction models in a mixed-ancestry population of South Africa
Source: Diabetol Metab Syndr. 2015 May 9;7:42. doi: 10.1186/s13098-015-0039-y (PMC4435909; doi:10.1186/s13098-015-0039-y)
Supplement: Additional file 1: Table S1. — Full equation for risk models to predict prevalent undiagnosed diabetes as applied to the Bellville South cohort. [file 13098_2015_39_MOESM1_ESM.doc]

## Additional file 1: Table S1: Full equation for risk models to predict prevalent undiagnosed diabetes as applied to the Bellville South cohort

| **Cambridge logistic regression diabetes risk model** [19] |
| --- |
| The probability of developing diabetes was calculated as exp(X)/(1 + exp(X)); where  X= -6.322 - 0.879 (if female, else 0) + 1.222 (if prescribed antihypertensive medication) + 2.191 (if prescribed steroids) + 0.063 × age in years + 0.699 (if 25 kg/m^2^ ≤ BMI ≤ 27.49 kg/m^2^) + 1.970 (if 27.5 kg/m^2^ ≤ BMI ≤ 29.99 kg/m^2^) + 2.518 (if BMI ≥ 30 kg/m^2^) + 0.728 (if parent or sibling has diabetes) + 0.753 (if parent and sibling has diabetes) - 0.218 (if an ex-smoker) + 0.855 (if a current smoker). |
| **Kuwaiti logistic regression diabetes risk model** [20] |
| The probability of developing diabetes was calculated as exp(X)/(1 + exp(X)); where  X= -5.018 + 0.979 (if a sibling had a history of diabetes, else 0) + 0.978 (if prescribed antihypertensive medication) + 1.315 (if age ≥ 35 years, else 0) + 1.930 (if the waist circumference ≥ 100cm, else 0). |
| **Omani logistic regression diabetes risk model** [21] |
| The probability of developing diabetes was calculated as exp(X)/(1 + exp(X)); where  X= -4.7 + 1.8 (if 40 years ≤ age ≤ 59 years) + 2.3 (if age ≥ 60 years) + 0.38 (if waist circumference ≥ 94cm in men and waist circumference ≥ 80cm in women) + 0.54 (if 25 kg/m^2^ ≤ BMI < 30 kg/m^2^) + 0.69 (if BMI ≥ 30 kg/m^2^) + 1.9 (if parental or sibling history of diabetes) + 0.73 (if if SBP≥140 and/or DBP≥90). |
| **Rotterdam logistic regression diabetes risk model** **1** [22] |
| The probability of developing diabetes was calculated as exp(X)/(1 + exp(X)); where  X= -3.02 + 0.19 (per 5 year increment from 55 years to >75) + 0.46 (if male, else 0) + 0.42 (if prescribed antihypertensive medication) + 0.51 (if BMI ≥ 30 kg/m^2^). |
| **Simplified Finnish logistic regression diabetes risk model** [23] |
| The probability of developing diabetes was calculated as exp(X)/(1 + exp(X)); where  X= -5.514 + 0.628 (if 45 years ≤ age ≤ 54 years) + 0.892 (if 55 years ≤ age ≤ 64 years) + 0.165 (if 25 kg/m^2^ ≤ BMI < 30 kg/m^2^) + 1.096 (if BMI > 30 kg/m^2^) + 0.857 (if 94 cm ≤ waist circumference < 102cm in men and 80cm ≤ waist circumference < 88 cm in women) + 1.350 (if waist circumference ≥ 102cm in men and waist circumference ≥ 88 cm in women) + 0.711 (if prescribed antihypertensive medication) + 2.139 (if a history of high blood glucose, assumed to be 0 for all participants due to the nature of this study). |

*BMI, body mass index; SBP, systolic blood pressure; DBP, dystolic blood pressure.
