# Supplementary material for: Independent external validation and comparison of prevalent diabetes risk prediction models in a mixed-ancestry population of South Africa
Source: Diabetol Metab Syndr. 2015 May 9;7:42. doi: 10.1186/s13098-015-0039-y (PMC4435909; doi:10.1186/s13098-015-0039-y)
Supplement: Additional file 2: Table S2. — Characteristics comparison of participants with valid and missing data. [file 13098_2015_39_MOESM2_ESM.doc]

## Additional file 2:Table S2: Characteristics comparison of participants with valid and missing data

| Characteristics | Valid (737) | Missing (346) | p-value |
| --- | --- | --- | --- |
| Male (%) | 157 (21.3) | 94 (27.2) | 0.012 |
| Age (years) | 51.2 (11.9) | 52.8 (18.3) | 0.104 |
| Body mass index (kg/m^2^) | 29.9 (7.3) | 29.6 (7.0) | 0.515 |
| Waist circumference (cm) | 96.4 (14.9) | 95.0 (16.2) | 0.147 |
| Hypertensive medication (%) | 251 (34.1) | 123 (35.6) | 0.182 |
| Smoking status (% smoking) | 323 (43.8) | 110 (31.8) | <0.001 |
| Systolic blood pressure (mmHg) | 122.8 (17.6) | 126.2 (23.1) | 0.009 |
| Diastolic blood pressure (mmHg) | 75.8 (11.5) | 76.2 (14.5) | 0.669 |
| Height (m) | 1.6 (0.1) | 1.6 (0.1) | 0.522 |
| Mother having diabetes (%) | 109 (14.8) | 15 (4.3) | <0.001 |
| Father having diabetes (%) | 58 (7.9) | 3 (0.9) | <0.001 |
| Sister having diabetes (%) | 92 (12.5) | 11 (3.2) | <0.001 |
| Brother having diabetes (%) | 58 (7.9) | 9 (2.6) | 0.001 |
| Fasting blood glucose (mmol/L) | 5.7 (1.9) | 5.5 (1.5) | 0.048 |
| HDL cholesterol (mmol/L) | 1.3 (0.4) | 1.3 (0.4) | 0.739 |
| Weight (kg) | 75.1 (17.9) | 74.7 (17.6) | 0.727 |
| Ever consumed alcohol (%) | 356 (48.3) | 88 (25.4) | <0.001 |
| Currently drinking (%) | 207 (28.1) | 65 (18.8) | 0.001 |
| Corticosteroid use (%) | 5 (0.7) | 7 (2.0) | 0.097 |
| Triglyceride (mmol/L) | 1.5 (1.0) | 1.4 (0.8) | 0.043 |

HDL, high-density lipoprotein.
