# Supplementary material for: Independent external validation and comparison of prevalent diabetes risk prediction models in a mixed-ancestry population of South Africa
Source: Diabetol Metab Syndr. 2015 May 9;7:42. doi: 10.1186/s13098-015-0039-y (PMC4435909; doi:10.1186/s13098-015-0039-y)
Supplement: Additional file 3: Table S3. — Discrimination values and 95 % confidence intervals for selected models and the comparison of the discrimination between each model, expressed using p-value (<0.05 significant). [file 13098_2015_39_MOESM3_ESM.doc]

## Additional file 3:Table S3: Discrimination values and 95% confidence intervals for selected models and the comparison of the discrimination between each model, expressed using p-value (<0.05 significant).

|  | C-statistic | Comparison discrimination | | | | |
| --- | --- | --- | --- | --- | --- | --- |
|  |  | Cambridge [19] | Kuwaiti [20] | Omani [21] | Rotterdam [22] | Finnish [23] |
| Cambridge [19] | 0.67 (0.62-0.72) | - | 0.689 | 0.458 | 0.066 | 0.734 |
| Kuwaiti [20] | 0.68 (0.63-0.73) | - | - | 0.292 | 0.109 | 0.397 |
| Omani [21] | 0.66 (0.61-0.70) | - | - | - | 0.605 | 0.735 |
| Rotterdam [22] | 0.64 (0.59-0.69) | - | - | - | - | 0.320 |
| Finnish [23] | 0.67 (0.62-0.71) | - | - | - | - | - |
